# Comparison of three quadratus lumborum block approaches for pediatric lower abdominal surgeries: a randomized controlled trial

**DOI:** 10.1016/j.bjane.2025.844683

**Published:** 2025-09-11

**Authors:** Arun SK, Ajeet Kumar, Amarjeet Kumar, Chandni Sinha, Abhyuday Kumar, Poonam Kumari, Bindey Kumar

**Affiliations:** a Department of Anesthesiology, All India Institute of Medical Sciences, Patna, India; b Department of Pediatric Surgery, All India Institute of Medical Sciences, Patna, India

**Keywords:** Anesthesia, Nerve block, Pain management, Pediatrics

## Abstract

**Background:**

Lower abdominal surgeries in the pediatric population are associated with significant post-operative pain. Regional anesthesia techniques including ilioinguinal nerve block, Transversus Abdominis Plane (TAP) block, and Quadratus Lumborum (QL) block have been explored for lower abdominal surgeries. This study compares the analgesic effect of three different approaches to quadratus lumborum block in pediatric patients undergoing lower abdominal surgeries.

**Methods:**

This randomized controlled trial included 120 pediatric patients aged between 1 and 7 years, scheduled for lower abdominal surgery under general anesthesia. Patients were randomized into 3 groups. Patients of Group A received QL block via anterior approach, Group L received QL block via lateral approach, and Group P received QL block via posterior approach. A volume of 0.5 mL.kg^-1^ of 0.375% ropivacaine was injected unilaterally for QL block in all patients. The primary outcome was 24hr postoperative fentanyl consumption. Secondary outcomes included intraoperative fentanyl use, postoperative pain scores, time to rescue analgesia and parental satisfaction.

**Results:**

Postoperative mean fentanyl consumption was significantly lower in Group A as compared to Group L (p < 0.001) and Group P (p < 0.011). Postoperative median FLACC scores were significantly lower (p < 0.05) in Group A in comparison to Group L and Group P in the early postoperative period. The parent satisfaction score was significantly higher (p < 0.05) in Group A.

**Conclusion:**

Anterior approach to QL block reduces postoperative analgesic consumption and provides longer duration analgesia with better parental satisfaction scores in comparison to lateral and posterior approaches in pediatric patients undergoing lower abdominal surgeries.

## Introduction

Lower abdominal surgeries in the pediatric population are among the most performed procedures and are associated with significant postoperative pain. Effective postoperative analgesia is therefore essential to ensure patient comfort. The current trend in pediatric pain management in anesthesia is moving beyond traditional opioid use, focusing instead on multimodal strategies to alleviate pain.^1^

Newer regional nerve blocks are being increasingly utilized, either to avoid the risks associated with neuraxial anesthesia or to minimize the side effects of opioids, such as hypotension, respiratory depression, pruritus, nausea, and vomiting.^2^, ^3^, ^4^, ^5^ Due to the potential complications of caudal blocks, including hypotension and urinary retention, alternative regional anesthesia techniques ‒ such as the Erector Spinae Plane (ESP) block, posterior Transversus Abdominis Plane (TAP) block, and Quadratus Lumborum (QL) block ‒ have been explored.^2^, ^3^, ^4^, ^5^ These blocks are typically performed under Ultrasound (US) guidance, which has enhanced their safety and utility in pediatric patients.^2^^,^^3^^,^^5^

The QL block is a posterior abdominal wall block first described by Blanco et al.^6^ It allows the spread of injected local anesthetic to the paravertebral space and has been used for abdominopelvic surgeries in pediatric and adult patients with good results.^7^, ^8^, ^9^ Earlier studies revealed that the quadratus lumborum block potentially results in extensive sensory blockade (T7–L2), with beneficial effects on both somatic and visceral pain.^10^ Various techniques of this block have been described, leading to differential spread of local anesthetic, and varied sensory and motor blockade. The QL muscle is surrounded by the Thoracolumbar Fascia (TLF), which consists of three distinct layers. The anterior layer blends laterally with the transversalis fascia and medially with the fascia of the psoas major. The middle layer lies between the QL and the erector spinae muscles, while the posterior layer is located posterior to the erector spinae. In the posterior approach, LA is deposited between the posterior surface of the QL and the TLF. In the lateral approach, LA is deposited between the muscle aponeurosis and the fascia at the lateral border of the QL. In the anterior (transmuscular) approach, LA is deposited between the anterior border of the QL and the psoas major (PM).^11^

Cadaver studies have shown that the anterior approach is characterized by cephalad migration into the Thoracic Paravertebral Space (TPVS) along the QL and PM muscles via a pathway posterior to arcuate ligaments.^12^ While previous studies have compared different regional anesthesia techniques, limited evidence exists on the comparative efficacy of anterior, lateral and posterior QL block approaches in pediatric patients. However, Kumar et al. compared three different approaches to QL block in adult patients who underwent inguinal hernia surgery.^13^ They found better postoperative analgesia in the anterior approach in comparison to the posterior or lateral approaches to the QL block. This is the first randomized controlled trial aimed at determining the optimal approach by assessing opioid consumption, pain scores and parental satisfaction.

We hypothesized that a pre-incisional anterior QL block would provide better postoperative analgesia, reduce 24-hour analgesic consumption and result in higher parental satisfaction compared to other approaches (posterior and lateral) in pediatric patients undergoing lower abdominal surgeries.

The primary objective of this study was to compare 24-hour postoperative fentanyl requirement among US guided anterior, posterior, and lateral approaches of the QL block in pediatric patients undergoing elective lower abdominal surgery. The secondary objectives included postoperative pain scores using the Faces, Leg, Activity, Cry, Consolability (FLACC) scale, duration of analgesia, intraoperative fentanyl consumption, parental satisfaction, and adverse effects like hematoma, vomiting, hypotension and others.

## Method

This double-blinded, randomized controlled trial was conducted at our tertiary care institute after obtaining Ethical committee clearance and registration with the Clinical Trial Registry of India (CTRI: 2020/02/023623) registration. This study was conducted prospectively over three years (April 2020 to February 2023). A total of 120 pediatric patients, aged 1 to 7 years with American Society of Anesthesiologists (ASA) physical status I and II, undergoing elective open lower abdominal surgery (orchidopexy, hernia repair, pyeloplasty) were included in this study. Patients whose parents refused to give consent, infection at the site of infection, and those suffering from coagulopathy, liver or kidney disorder were excluded from this study. Patients meeting the inclusion criteria were randomized into 3 groups (40 patients each). Simple randomization was done by the co-PI (CS) using the online software (Open Epi software version 3.01, Atlanta, GA, USA). The allocation sequence was concealed in sequentially numbered opaque, sealed envelopes that were opened by the primary surgeon on the day of surgery.

Patients in Group A received QL block via the anterior approach, Group L via the lateral approach, and Group P via the posterior approach. A volume of 0.5 mL.kg^-1^ of 0.375% ropivacaine was injected unilaterally for QL block in all patients. Written and informed consent for publication was obtained from the parents of every patient. This study was conducted in accordance with the principles of the Declaration of Helsinki.

All patients received oral midazolam (0.5 mg.kg^-1^) one hour before shifting to the Operating Room (OR). Upon arrival in the OR, standard monitors including Heart Rate (HR), Non-Invasive Blood Pressure (NIBP), Electrocardiogram (ECG), Oxygen Saturation (SpO_2_) were applied and recorded. Anesthesia was induced with an injection of fentanyl 2 mcg.kg^-1^, propofol 2 mg.kg^-1^, and atracurium 0.5 mg.kg^-1^. This was followed by trachea intubation with an appropriate-size endotracheal tube. Anesthesia was maintained with 2% sevoflurane in 50% oxygen. Hemodynamic parameters (HR and Mean Arterial Pressure (MAP) were recorded every 5 minutes till the end of surgery. Ultrasound-guided QL block was performed after induction of anesthesia with patients placed in the lateral decubitus position. All blocks were performed by trained anesthesiologists with over 7 years of experience in administering US-guided blocks. These anesthesiologists were not involved in data collection, which was performed by Operating Room (OR) residents. Postoperative assessments were conducted by trained pain nurses blinded to the intraoperative interventions.

Technique of QL block: All QL blocks were performed in the lateral position. A high-frequency linear probe (Ultrasound machine Edge II, Fujifilm Sonosite, Inc., Bothell, WA, United States) was used to visualize the structures and a 22G, 80 mm echogenic needle (Sonoplex needles, Pajunk, Germany) was inserted to deposit the drug.

**Figure 1:**
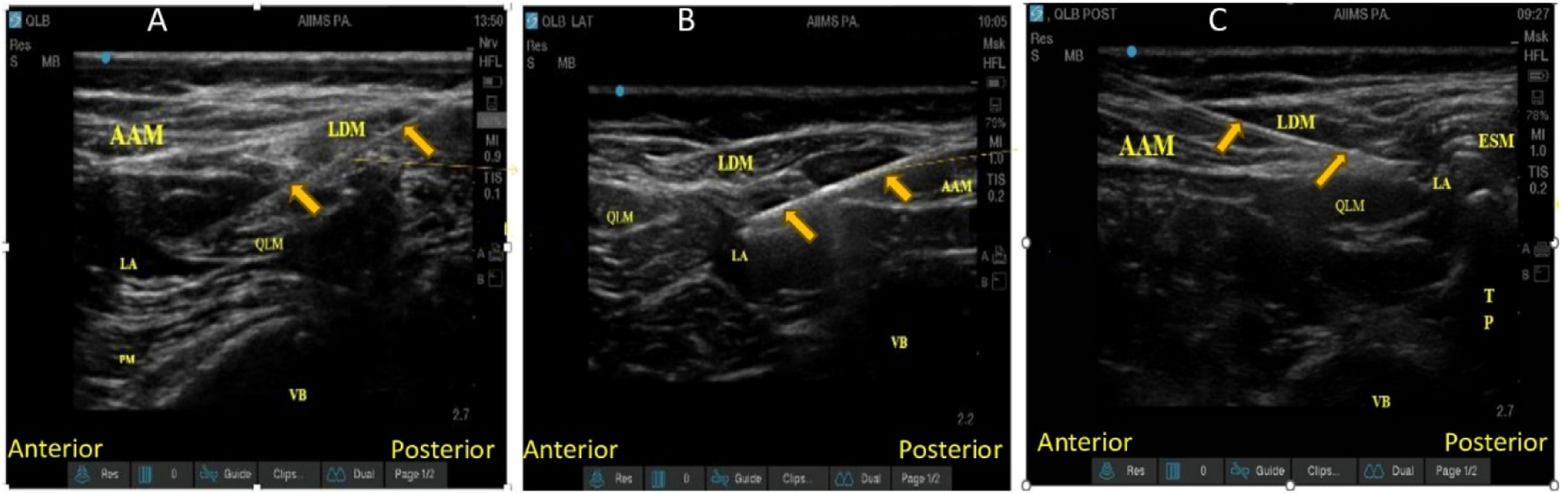
Sonoanatomy of QL block, Panel A: Anterior approach; Panel B: Lateral approach; Panel C: Posterior approach. AAM, Anterior Abdominal Muscle; ESM, Erector Spinae Muscle; LA, Local Anesthetic; LDM, Latissimus Dorsi Muscle; QLM, Quadratus Lumborum Muscle; VB, Vertebral Body.

Anterior QL block: The probe was placed above the iliac crest, and Petit’s triangle was identified. The three abdominal muscles (i.e., the external oblique, internal oblique, and transversus abdominus muscles) were identified and followed posteriorly until the layers of the Thoracolumbar Fascia (TLF) appeared as a bright hyper-echogenic line. The needle was inserted in-plane along the posterior edge of the probe in anteromedial direction (Fig. 1, panel A) targeting the plane between the quadratus lumborum and psoas major muscle. After confirming the correct needle tip position and negative aspiration for blood, ropivacaine was injected.

Lateral QL block: The probe was placed in the axial plane in the mid-axillary line, and moved posteriorly until the posterior aponeurosis of the transversus abdominis muscle became visible. The needle was inserted from the anterior and advanced until the needle tip just penetrated the posterior aponeurosis of the transversus abdominis muscle (Fig. 1, panel B). Local anesthetic was injected between the aponeurosis and the fascia at the lateral margin of the QL muscle. Posterior QL block: With the patient in the lateral position, the probe was again placed in the axial plane at the mid-axillary line and moved posteriorly to identify the posterior border of the QL muscle. The needle tip was placed between QL and the erector spinae muscle (Fig. 1, panel C).

At the end of surgery, all patients received a diclofenac suppository (1 mg.kg^-1^) and intravenous paracetamol (15 mg.kg^-1^) every 8 hours during the postoperative period. Fentanyl (1 mcg.kg^-1^) was administered intraoperatively and postoperatively in response to a 20% increase in HR or MAP from baseline or if the FLACC score exceeded 4.

Complications like postoperative nausea and vomiting, motor weakness, or block site occurring during the procedure were documented. Patients were extubated after they were awake and generating adequate tidal volume. Postoperative pain was assessed using a FLACC (Face, Legs, Activity, Cry, Consolability) scale at 30 minutes, 2, 4, 8, 12, and 24 hours. IV Fentanyl 1 mcg.kg^-1^ was administered till 24 hours if the FLACC was more than 4. The time to first rescue analgesic requirements in the postoperative period was documented. Parental satisfaction with pain management was rated on a 10-point Likert scale (where 0 represented the lowest and 10 the highest level of satisfaction).^14^

An online calculator (www.clincalc.com) was used to calculate the sample size and power analysis using the Neyman-Pearson approach based on a pilot study done on 18 pediatric patients receiving anterior, lateral, and posterior approaches of QL block. The 24-hour postoperative fentanyl requirement was found to be (17 ± 9 mcg), (25 ± 12 mcg), and (22 ± 12 mcg), respectively, in the anterior, lateral, and posterior approaches of the QL block. Assuming a mean fentanyl difference of 8 mcg between the groups, a standard deviation of 12, a power of 80% and alpha as 0.05, the sample size came out to be 35 in each group. Considering 15% dropouts, we included a total of 120 patients (40 in each group).

Data were entered into Microsoft Excel and analyzed in IBM SPSS software version 23. The normality of the data was tested using the Shapiro-Wilk test. Normalcy of data was checked using the Shapiro-Wilk test. Continuous quantitative variables are presented as mean ± Standard Deviation (SD) and the intergroup comparisons between the three groups were analyzed by Analysis of Variance (ANOVA) with post-hoc analysis. Quantitative discrete data like FLACC score, time required for first rescue analgesia, and total analgesic consumption were presented as median (IQR) as all the data were not normally distributed when tested using the Shapiro-Wilk test. The Kruskal-Wallis test with pairwise comparisons was applied for comparisons between anterior, lateral, and posterior blocks for the pain scores, time required for first rescue analgesia, and total opioid consumption. Bonferroni corrections were applied for multiple pairwise comparisons between the groups and p-values < 0.0167 were taken as significant. All other comparison levels of p-value < 0.05 were taken as significant.

## Results

**Figure 2:**
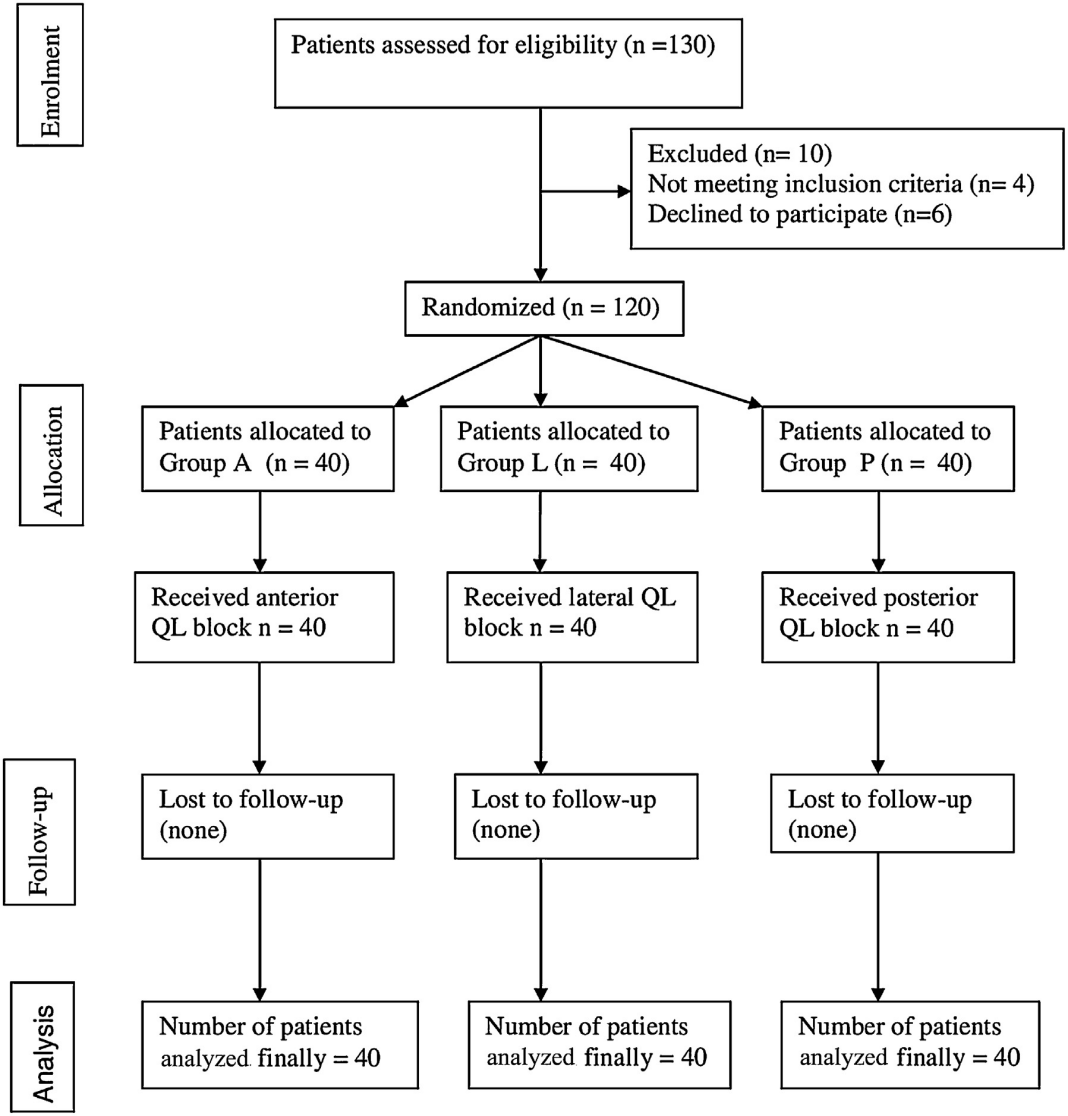
Consort flow diagram of study population.

A total of 130 patients were assessed for eligibility, of whom 10 were excluded (4 did not meet the inclusion criteria and 6 declined to participate). The remaining 120 patients were randomly assigned to three groups and completed the study protocol (Fig. 2). The surgical and demographic characteristics were similar in all 3 groups (Table 1). Postoperative mean fentanyl requirements were lower in Group A (15.0 ± 7.47), than in Group L (23.80 ± 9.56), and in Group P (20.15 ± 9.59) (Table 2). Post-hoc analysis showed significant differences when Group A was compared with Group L and Group P, while differences were insignificant between Group L and Group P (Table 3). Intraoperative mean fentanyl requirement was also lower in Group A (18.48 ± 6.46), in comparison to Group L and Group P (20.0 ± 5.20 and 20.0 ± 8.98 respectively), although differences among groups were insignificant (p > 0.05) (Table 2). Post-hoc analysis also showed insignificant differences among groups (Table 3).

**Table 1:** Demographic and surgical characteristics.

Characteristics	Group A (n = 40)	Group L (n = 40)	Group P (n = 40)	p-value
Age (years)^a^	3.73 ± 1.48	3.30 ± 1.45	2.98 ± 1.49	0.086
Weight (Kg)^a^	16.65 ± 4.28	17.65 ± 5.56	16.10 ± 5.81	0.413
Duration of surgical procedure (mins)^a^	55.0 ± 19.35	55.50 ± 19.93	61.50 ± 18.12	0.244
Types of surgery^b^: Hernia / Orchidopexy / Pyeloplasty	13/13/14	10/14/16	16/10/14	0.690

a Analysis of Variance (ANOVA), data expressed as mean and Standard Deviation (SD).

b Chi-Square test, data expressed as frequency.

**Table 2:** Comparison of intraoperative and postoperative opioid consumption, time to first rescue analgesia and postoperative parental satisfaction.

Parameter	Group A (n = 40)	Group L (n = 40)	Group P (n = 40)	p
Intraoperative fentanyl requirements (mcg)^a^	18.48 ± 6.46	20.00 ± 5.20	20.00 ± 8.98	0.538
Postoperative fentanyl consumption (mcg)^a^	15.00 ± 7.47	23.80 ± 9.56	20.15 ± 9.59	0.001
Time to 1^st^ rescue analgesia (hrs)^b^	20 (16‒24)	13 (10‒16)	8 (7‒16)	0.001
Parental satisfaction score^a^	8.50 ± 0.55	7.43 ± 0.64	8.00 ± 1.01	0.001

a Analysis of Variance (ANOVA), data expressed as mean and Standard Deviation (SD).

b Kruskal-Wallis test, data expressed as median and interquartile range (IQR), p < 0.05: significant.

**Table 3:** Post-hoc analysis of intraoperative and postoperative opioid consumption, time to 1^st^ rescue analgesia and postoperative parental satisfaction between the groups.

Variables	Intergroup comparison	Mean difference	95% CI	p-value
Intraoperative fentanyl requirements (mcg)	Group A & Group L	-1.52	-4.65 to 1.60	0.336
	Group A & Group P	-1.52	-4.65 to 1.60	0.336
	Group L & Group P	0.00	-3.13 to 3.13	1.000
Postoperative fentanyl consumed (mcg)	Group A & Group L	-8.80	-12.75 to -4.85	0.001^a^
	Group A & Group P	-5.15	-9.10 to -1.20	0.011^a^
	Group L & Group P	3.65	-0.30 to 7.60	0.070
Time to 1^st^ rescue analgesia (hrs)	Group A & Group L	5.17	2.79 to 7.56	0.001^a^
	Group A & Group P	7.15	4.76 to 9.54	0.001^a^
	Group L & Group P	1.975	-0.41 to 4.36	0.104
Parental satisfaction score	Group A & Group L	1.07	0.74 to 1.41	0.001^a^
	Group A & Group P	0.50	0.16 to 0.84	0.004^a^
	Group L & Group P	-0.57	-0.91 to -0.24	0.001^a^

CI, Confidence Interval.

a p-value < 0.0167 is taken as statistically significant.

Median time to first rescue analgesic requirements was significantly prolonged in Group A in comparison to Group L and Group P (p < 0.05) (Table 2). Post-hoc analysis showed significant differences when Group A was compared with Group L and Group P, while differences were insignificant between Group L and Group P (Table 3).

Median FLACC scores showed variable results among the groups at different time points. It was significantly lower in Group A in comparison to Group L and Group P during the early postoperative period (at 4 hours p < 0.05), while the differences were insignificant at 8 hours, at 12 hours and at 24 hours after surgery (p > 0.05) (Table 4).

**Table 4:** Comparison of median FLACC “Scores”.

FLACC score (rest)	Group A (n = 40)	Group L (n = 40)	Group P (n = 40)	p-value
30 mins	3 (2‒4)	4 (3‒5)	4 (3‒6)	0.001
2h	3 (2‒4)	4 (2‒4)	4 (4‒5)	0.001
4h	3 (1‒5)	3 (2‒4)	4 (3‒6)	0.003
8h	3 (1‒4)	3 (3‒4)	3 (1‒5)	0.320
12h	1.5 (0‒3)	2 (2‒3)	3 (2‒4)	0.065
24h	1 (0‒3)	1 (0‒2)	2 (1‒2)	0.800

Kruskal-Wallis test, data expressed as median and Interquartile Range (IQR), p < 0.05: significant, FLACC, Faces, Leg, Activity, Cry, “Consolability”.

The parent satisfaction score was significantly higher in Group A (8.5 ± 0.55) compared to the other two groups, Group L and Group P, (7.43 ± 0.64 and 8.0 ± 1.01, respectively; p < 0.05) (Table 2). Post-hoc analysis also showed significant differences among groups (Table 3). There were no complications in any of the groups.

## Discussion

Our findings indicate that the anterior QL block is superior to the lateral and posterior approaches in terms of postoperative analgesic outcomes. Post-hoc analysis also showed a significantly prolonged duration of analgesia, decreased requirement for postoperative fentanyl, and higher parent satisfaction in the anterior QL block group. FLACC scores were significantly lower in Group A compared to Group L and Group P during the early postoperative period, up to 4 hours. However, the difference in median FLACC scores may not be clinically significant, as the difference in medians was not more than 2 points. This discrepancy might be attributed to the rescue dose of fentanyl administered during the postoperative period, which could have influenced the FLACC scores. The median duration of analgesia in Group A was significantly longer compared to Group L and Group P (20 hours vs. 13 hours vs. 8 hours, respectively), which appears to be clinically significant as well.

Lower abdominal surgeries are common in pediatric patients, and inadequate pain control can lead to complications such as delayed recovery, poor patient satisfaction, and the development of chronic pain syndromes. The Quadratus Lumborum (QL) block provides sensory analgesia to the inguinal region by consistently blocking both the iliohypogastric and ilioinguinal nerves (root values L1 and L2), owing to its wide dermatomal coverage (T7–L2).^10^

The proposed hypothesis for superior analgesia with the anterior QL block is that it allows a more extensive spread of Local Anesthetic (LA) to the lumbar nerve roots and their branches, as well as to the thoracic paravertebral space, thereby providing both somatic and visceral analgesia.^15^^,^^16^ In contrast, the posterior QL block limits drug spread primarily to the middle thoracolumbar fascia and intertransverse area.^6^ The lateral QL block achieves its effect via spread along the transversus abdominis plane and into the subcutaneous tissue.^17^

These findings are supported by a cadaveric dye study conducted by Elsharkawy et al,^18^ in which dye spread widely into the thoracic paravertebral space (T9–T12), staining the iliohypogastric and ilioinguinal nerves (L1), and the subcostal nerve in the anterior approach, thereby providing broader coverage and more effective sensory blockade.

Sato et al., in a study involving pediatric patients, reported that the QL block was superior to both the Transversus Abdominis Plane (TAP) block and caudal blocks in terms of pain scores, patient satisfaction, and the number of patients requiring rescue analgesia.^19^ Similarly, Aksu and Gurkan demonstrated that the QL block was effective for pediatric day-care hernia surgeries and outperformed the TAP block in terms of analgesic efficacy.^20^

Since its initial description in 2007, the QL block has evolved, with three commonly practiced approaches. Ahmed et al. compared the anterior and posterior approaches in patients undergoing unilateral inguinal hernia repair.^21^ They found that patients receiving the anterior approach had significantly longer-lasting analgesia compared to those who received the posterior approach.

Despite these findings, literature describing the use of QL blocks in children for postoperative analgesia across various surgeries remains limited.^22^, ^23^, ^24^ To the best of our knowledge, no randomized studies have compared all three approaches ‒ anterior, posterior, and lateral ‒ in the pediatric population. However, El Malla et al.^25^ compared the anterior and posterior approaches and found that the anterior QL block provided a better analgesic profile, with significantly reduced postoperative morphine consumption, longer analgesic duration, and lower pain scores, without any adverse effects, in pediatric patients undergoing laparoscopic inguinal hernia repair.

Our findings align with these results, showing reduced requirements for rescue analgesia during both intraoperative and postoperative periods, improved pain scores, and prolonged analgesia with the anterior approach. This approach also yielded higher parental satisfaction compared to the lateral and posterior approaches.

In contrast, a study by Ahuja et al.^26^ found that a single-shot anterior QL block offered no significant advantage over no block in pediatric patients undergoing unilateral inguinal hernia surgery under Subarachnoid Block (SAB). This may be due to various factors affecting the spread of local anesthetic, including anatomical variations, the path of least resistance, injection speed, and the volume of anesthetic administered.

The anterior approach was first described by Borglum et al. An MRI study conducted one-hour post-injection demonstrated that the LA had spread cephalad to reach the thoracic paravertebral space. This spread was attributed to the shared embryological origin and insertion of the psoas major and quadratus lumborum muscles within the thoracic cage. These findings were later confirmed by additional cadaveric studies.

There have been reports of lower limb weakness due to the spread of LA to the lumbar plexus. Although we did not specifically assess muscle strength due to the young age of our participants, we did not observe any visible signs of lower limb weakness.^27^ Additionally, no complications related to QL block were encountered in our study. Despite being a deep block, which can be associated with risks such as retroperitoneal hematoma, organ injury, or local anesthetic toxicity,^28^ no adverse events occurred. However, spread to the paravertebral space can, in some cases, lead to hypotension and bradycardia.

This study has several limitations. First, the inclusion of heterogeneous surgical procedures with distinct pain profiles could have influenced analgesic consumption and parental satisfaction outcomes. A stratified randomization or subgroup analysis may have mitigated this issue. Second, although experienced anesthesiologists performed the blocks, subtle visual or tactile clues may have compromised blinding. Third, due to the pediatric population, no objective assessment of motor weakness was conducted, despite potential lumbar plexus spread, especially with the anterior approach. Lastly, this study employed a single-shot block; continuous catheter techniques may yield different analgesic profiles and warrant further investigation.

## Data availability statement

The data sets generated during and/or analyzed during the current study are available in the SciELO Data repository - https://doi.org/10.48331/SCIELODATA.Q22LU0. Any additional data will be made available upon reasonable request to the corresponding author.

## Conclusion

In this randomized controlled trial, the anterior approach to the quadratus lumborum block was associated with lower postoperative opioid consumption, longer duration of analgesia, and higher parental satisfaction compared to lateral and posterior approaches in pediatric patients undergoing lower abdominal surgery. Despite these findings, further multicenter studies with larger and more homogeneous populations are warranted to confirm these results and refine clinical guidelines for QL block in pediatrics.

## Conflicts of interest

The authors declare no conflicts of interest.
